# Probing the Pre-diagnostic Phase of Parkinson's Disease in Population-Based Studies

**DOI:** 10.3389/fneur.2021.702502

**Published:** 2021-07-01

**Authors:** Lisanne J. Dommershuijsen, Agnita J. W. Boon, M. Kamran Ikram

**Affiliations:** ^1^Department of Epidemiology, Erasmus MC University Medical Center, Rotterdam, Netherlands; ^2^Department of Neurology, Erasmus MC University Medical Center, Rotterdam, Netherlands

**Keywords:** heterogeneity, pre-diagnostic Parkinson's disease, prodromal Parkinson's disease, population-based, epidemiology, prevention trials, prediction, target trial emulation

## Abstract

Parkinson's disease covers a wide spectrum of symptoms, ranging from early non-motor symptoms to the characteristic bradykinesia, tremor and rigidity. Although differences in the symptomatology of Parkinson's disease are increasingly recognized, there is still a lack of insight into the heterogeneity of the pre-diagnostic phase of Parkinson's disease. In this perspective, we highlight three aspects regarding the role of population-based studies in providing new insights into the heterogeneity of pre-diagnostic Parkinson's disease. First we describe several specific advantages of population-based cohort studies, including the design which overcomes some common biases, the broad data collection and the high external validity. Second, we draw a parallel with the field of Alzheimer's disease to provide future directions to uncover the heterogeneity of pre-diagnostic Parkinson's disease. Finally, we anticipate on the emergence of prevention and disease-modification trials and the potential role of population-based studies herein. In the coming years, bridging gaps between study designs will be essential to make vital advances in elucidating the heterogeneity of pre-diagnostic Parkinson's disease.

## Introduction

Parkinson's disease (PD) covers a wide spectrum of symptoms, ranging from early non-motor symptoms, such as constipation, REM-sleep behavior disorder (RBD) and hyposmia (1, 2), to the characteristic motor symptoms bradykinesia, tremor and rigidity (3). A similar appearing Parkinson syndrome can result from many different causes and even one specific cause can bring about heterogeneous symptomatology (4). Although the diversity of clinical PD is increasingly being recognized (5), there is still a lack of insight into the heterogeneity of the pre-diagnostic phase of PD (6, 7).

Studying the heterogeneous nature of pre-diagnostic PD is challenging but essential to unravel etiology and to recognize symptoms and modify disease progression in an early phase. Population-based cohort studies are particularly suitable to gain insight into pre-diagnostic PD (8). In this perspective, we will discuss some unique design advantages of population-based cohort studies for elucidating preclinical PD. In addition, we will elaborate on lessons learned from population-based studies in the field of Alzheimer's disease (AD) to provide future directions for understanding the diversity of risk factors and prodromal symptoms of PD. Finally, we will describe how population-based studies can be used optimally on the way toward prevention and disease-modification trials.

## Phases of Pre-Diagnostic Parkinson's Disease

The Movement Disorders Society (MDS) task force on the definition of PD has suggested to divide early PD into three phases: preclinical, prodromal and clinical PD (9, 10). Besides these three phases, even an earlier risk phase can be distinguished (2). The risk phase, the preclinical phase and the prodromal phase together are considered pre-diagnostic PD (2, 11). Clinical and case-control studies have thus far provided important insights into clinical PD and have uncovered initial clues regarding pre-diagnostic PD (2, 12). However, population-based studies are necessary to better understand the different phases of the pre-diagnostic period of PD (8).

The first phase of pre-diagnostic PD is also known as the *risk phase* (2, 13, 14). In this phase, people can be exposed to several risk factors and pathophysiological processes may initiate, but no PD-related pathology is present yet. Case-control studies have increased our understanding of the genetic risk of PD, including the identification of novel risk loci (15). The case-control design is especially valuable for studying genetic risk factors because these per definition precede the PD diagnosis. However, PD risk is also importantly influenced by a combination of environmental and lifestyle factors which usually have a long latency period and accumulate during life course (16, 17). Studying these type of risk factors in case-control studies yields several methodological challenges and potential sources of bias (18). Additional research by population-based studies is thus indispensable to obtain further insight into the risk phase of PD.

The second phase of pre-diagnostic PD is the *preclinical phase*, during which the pathology has initiated and biomarkers can be found that are suggestive of PD. During the preclinical phase, however, no symptoms of disease have yet arisen (2). Currently, the predictive ability of preclinical biomarkers for future PD diagnosis is limited, but research is ongoing (4, 11, 19, 20). Promising biomarkers for early detection and diagnosis of PD include α-synuclein measurements in the cerebrospinal fluid, blood and peripheral tissue (11, 19, 20). Furthermore, as dopamine loss occurs steadily throughout pre-diagnostic PD (2, 21, 22), dopamine transporter scanning might be used as an imaging marker of the early phases of PD (11, 20). Interestingly, one study found a reduced striatal uptake in 44% of asymptomatic mutation carriers of the LRRK2 mutation G2019S1 (23), a mutation with incomplete penetrance for PD (24, 25). Only two carriers (14%) in this study had hyposmia and the motor score was comparable to non-carriers (23). This might reflect that dopamine transporter abnormalities could already be found in the preclinical phase of PD. However, a more recent study found only 11% of non-manifest LRRK2 carriers to have a dopamine transporter deficit, even though their clinical characteristics already differed significantly from healthy controls (26). The value of this marker in preclinical PD thus requires further consideration. Although studies in clinical populations, such as people with RBD, can provide some initial insights into possible biomarkers for early PD (27–30), only large population-based studies can determine the ability of these markers to identify preclinical PD.

The final phase of pre-diagnostic PD is the *prodromal phase*. This phase can last over a decade and is mainly characterized by non-motor symptoms (1, 2), although recent studies have also shown subtle motor deficits years before the diagnosis (1, 2, 31). Symptoms in the prodromal phase of PD are often not yet recognized as belonging to PD because of their low specificity (2). RBD, however, is a highly specific symptom of prodromal α-synucleinopathies (PD, dementia with Lewy bodies and multiple system atrophy) (2, 32). The relation between RBD and α-synucleinopathies was first described in clinical case studies (33) and has since been confirmed by many other clinical studies with long-term follow-up (32, 34). However, studies recruiting patients in sleep clinics will not be generalizable to all people with RBD since RBD is generally underdiagnosed (32, 35, 36). Therefore, confirmation of the association between RBD and α-synucleinopathies in population-based studies is needed (37).

## Importance of Population-Based Studies in Pre-Diagnostic Parkinson's Disease

Studying risk factors, preclinical biomarkers and prodromal symptoms in case-control studies is methodologically challenging. For these type of study questions regarding pre-diagnostic PD, the design of population-based cohort studies offers several important advantages.

First, prospective cohort studies surmount some common biases in case-control studies (13, 18, 38). Recall bias, a type of differential misclassification bias, is an important issue in case-control studies, which prospective cohort studies overcome (18). This bias is especially relevant to PD because of the very long prodromal period in which symptoms, such as constipation (2), can appear that could be more often recognized by cases than controls (13). In addition, case-control studies often suffer from selection bias at study entry (13, 18, 39). Selection bias could, for instance, occur in case-controls studies investigating the association of diet with PD (40). If controls are selected on the basis of an advertisement about research on nutrition, usually this will attract people who have an interest in this topic and who already have a healthy diet (18, 41). As such, the exposure of interest, diet, will be associated with study participation and with having the outcome PD, which results in selection bias. Selection bias in case-control studies can also occur in the selection of cases, when the exposure is related to survival and only cases who survive are included (42). This bias is often referred to as survivor bias (42). Survivor bias could importantly distort the findings of case-control studies, if this is not taken into account appropriately. In population-based cohort studies, selection bias can also be an issue, especially due to selective loss to follow-up or competing risks (18, 43). Fortunately, frameworks are now being developed for the appropriate adjustment for this possible source of bias in cohort studies (44).

Second, population-based studies often routinely collect data on many population characteristics. These variables can be used to extensively adjust for confounding bias. If we again take the example of the association between diet and PD, appropriate adjustment for smoking is necessary because smoking behavior is associated with both diet and PD risk and thus could be a confounder in this association (39, 45). In addition, broad data collection provides the opportunity to study the interdependence of markers of pre-diagnostic PD (46, 47) and to combine multiple markers in a prediction algorithm for PD (48–54), such as the PREDICT-PD algorithm (55, 56) and the naïve Bayesian classifier approach of the MDS research criteria for prodromal PD (54). Finally, the large datasets of population-based studies offer possibilities to study the combined effect of multiple risk factors on PD, as well as on clusters of chronic diseases (57), which is essential given that many risk factors and chronic diseases co-occur.

Third, contrary to clinical studies, the generalizability of population-based studies is usually high because of limited selection criteria at baseline (58). This difference in external validity might explain some common discrepancies in findings between clinical studies and population-based studies. As an illustration, while specialized clinics found a substantial increased likelihood for PD in people with neurogenic orthostatic hypotension (54, 59, 60), in the population-based Rotterdam study we previously found no significant association between neurogenic orthostatic hypotension and PD (61). The high external validity of population-based studies also offers several possibilities, for example to study the prevalence and trajectories of symptoms in pre-diagnostic PD, which is necessary to determine the predictive ability of these symptoms (8, 31). For example, hyposmia is a common symptom of prodromal PD (2), but is also highly prevalent in the general older population (62). Estimating the prevalence of hyposmia in the general population thus provides imperative information on the usefulness of this symptom to predict PD. Lastly, trends over time in PD incidence and mortality can easily be studied in population-based studies because of the often standardized criteria for determining PD (63).

Currently, several population-based studies are ongoing with a focus on PD (2, 8, 13, 14). Some of these studies are specifically designed for the outcome PD, such as the PREDICT-PD (55) and the PRIPS study (64), which have the advantage of choosing specific measures of importance to PD. Other studies, such as the Rotterdam Study (65) and the Honolulu-Asia Aging Study (HAAS) (66), take PD into account as one of the outcomes. The advantage of this approach is the often larger available sample, but this comes at the price of less PD-specific measurements.

## Learning from Population-Based Studies in Alzheimer's Disease

Over the last decades, the AD field has made significant progress in the discovery of risk factors and preclinical biomarkers (67). Population-based studies have played an important role in these advances. For example, population-based studies have provided crucial insights into the role of vascular factors in the etiology of AD (68, 69). Taking a broader perspective and recognizing similarities between PD and AD will help to make further progress in PD research (70–72).

PD and AD are both complex diseases in which modifiable risk factors play an important role (67). The AD field has shown the value of studying the interplay between different risk factors. A recent meta-analysis, for instance, has illustrated a dose-response relationship between the number of modifiable risk factors and the risk of dementia (73). Additionally, several studies have calculated the population-attributable fraction for each modifiable risk factor separately as well as for all risk factors combined, which shows the prevention potential of improving lifestyle (67, 74). In the AD field, moreover, a greater emphasis is placed on life course determinants and differences in risk factors in early-, mid, and late-life (67, 68, 70). Such a holistic approach to modifiable risk factors in population-based studies could help PD research in moving toward prevention and disease-modification trials.

Furthermore, both PD and AD require easy-to-measure preclinical biomarkers for earlier disease recognition and recruitment of participants in trials (4, 11, 19, 75–78). AD research has shown the benefit of considering distinct biomarkers at different disease phases (78, 79) and of creating panels of multiple biomarkers (78, 79). This is especially relevant to PD, given the heterogeneity of the disease (11, 19, 20, 72, 75, 80) and the diverse pathophysiological mechanisms at play at different disease stages (4, 20, 75). Population-based cohort studies provide opportunities to study biomarkers for PD at very early stages (75, 78, 81).

Finally, both the PD and AD field have recognized the importance of harmonization of measurements and data sharing (47, 70, 80, 82). Data sharing enables population-based studies to study small effects, to perform subgroup analyses and to replicate findings (47, 70, 80, 82). Harmonization of measurements and subsequent data pooling is of particular importance in PD research given the low disease incidence, which requires a large study population and long follow-up period for sufficient study power (14, 18, 83). Good examples of harmonization of measurements and data sharing include the Parkinson's Progression Markers Initiative (PPMI) (20, 84), including more than 30 centers across different countries, the Global Parkinson's Genetics Program (GP2), including 150,000 volunteers around the world (85), and the Alzheimers Cohorts Consortium, including nine population-based cohort studies in the United States and Europe (86). In addition to data sharing, several initiatives have been launched in the AD field to promote knowledge and expertise sharing (47, 87), including Methods for Longitudinal Studies in Dementia (MELODEM) (88) and Alzheimer's Association Professional Interest Areas (PIAs) (70). Both initiatives promote knowledge sharing, either through recommendations for dealing with common methodological difficulties, or through networking, mentoring and collaborations. In the PD field, very recently the Dutch Parkinson Alliance was founded (89), which includes an association aiming to connect PD researchers (90). Such initiatives help to adopt novel and more advanced methodologies and to avoid common pitfalls in research (70).

## Studying the Heterogeneity of Pre-Diagnostic Parkinson's Disease in Population-Based Studies

The pre-diagnostic phase of PD is heterogeneous with regards to risk factors, biomarkers and prodromal symptoms (2, 4, 12). Taking into account this heterogeneity in population-based studies is essential because a lack of insight into interactions between risk factors and subgroup differences in prodromal symptoms precludes our understanding of the different pathophysiological processes involved in PD and hinders early disease recognition.

Important developments regarding the complex interaction between risk factors of PD stem from studies on gene-environment interactions (12, 91–94). Studying the interplay between genetics and the environment provides insights into why only certain individuals exposed to environmental risk factors develop PD. The interaction between genetic mutations and pesticides is a major field of study herein (91, 92). A 2012 study, for example, showed that the association between paraquat exposure and PD was seven times greater in men with homozygous deletions of GSTT1, a gene involved in the metabolism of chemical substances, than in men with functional GSTT1 (95). Further application of these methods in population-based studies on environmental and lifestyle risk factors is warranted.

Heterogeneity in the prodromal phase of PD is currently understudied (12, 96). A recent review article has highlighted the importance of acknowledging heterogeneity in prodromal PD for early disease recognition and targeted neuroprotective interventions (96). Several subtypes in the prodromal phase were mentioned in this review, including RBD subtypes, brain-first and body-first subtypes, genetic subtypes and biological subtypes. With regards to differences in RBD subtypes, RBD in the prodromal phase seems to predispose to a more malignant PD subtype, especially regarding non-motor symptoms (97–99). The brain-first vs. body-first subtypes, suggesting that α-synuclein pathology originates either in the brain itself or in the autonomic nervous system, might explain the difference in the sequence of the occurrence of symptoms in the prodromal phase (96, 100, 101). These subtypes of prodromal PD require further investigation in longitudinal studies (96). Recognition of these different subtypes in the prodromal phase is important, but acknowledging heterogeneity in basic patient characteristics, for instance by sex and ethnicity, is also essential (6, 47). However, the current MDS criteria for prodromal PD do not distinguish prodromal symptoms between men and women or people of different ethnicities (54). Nevertheless, some initial studies have shown important differences in prodromal symptoms between men and women (6, 102–105). One of these studies reported more sexual dysfunction, memory complaints and dream re-enactment in men and more unexplained weight change and anxiety in women (102). In addition, symptoms in the prodromal as well as the clinical phase of PD have been suggested to differ between people of different ethnicities (103–105), but evidence regarding the prodromal phase is very limited. Some initiatives have been launched to increase our understanding of PD in underrepresented areas (106–109). However, for instance in Africa the conducted studies are still mainly door-to-door surveys or case-control studies (106, 110, 111) and population-based cohort studies are missing (63, 106–109). Studies in underrepresented areas are needed to understand variations in occurrence and phenotype of PD in different populations and to elucidate complex interactions between risk factors (4, 63, 104, 105, 107, 108). More diverse study populations, as well as more elaborate stratification by population characteristics, symptoms and biomarkers are desired (20).

## The Role of Population-Based Studies in Moving Toward Trials

### From Population-Based Studies to Trials

Input from observational studies is necessary to guide future prevention and disease-modification trials in recruiting at-risk populations, choosing preventive interventions and selecting outcome measures (8, 20, 112). Population-based studies provide especially important insights into the first two categories.

First, selecting an appropriate study population is pivotal for the success of future prevention and disease-modification trials (8). Currently, multiple enriched risk cohort studies are ongoing, including the Tübinger evaluation of Risk factors for Early detection of NeuroDegeneration (TREND) study, the Parkinson Associated Risk Study (PARS), the Parkinson's Progression Markers Initiative prodromal cohort (PPMI) and the Oxford Parkinson's Disease Centre (OPDC), which provide insight into early phases of PD and create platforms to recruit subjects for trials (8, 11, 53). These studies include various populations with a high risk of PD, such as asymptomatic mutation carriers, people with RBD or people with composite prodromal features. Each of these study populations have specific advantages and disadvantages for future trial recruitment. For instance, the advantage of recruiting individuals with prodromal symptoms is the short lead time to PD, whereas this is simultaneously a disadvantage since the disease might be too advanced for trials to be successful (11). Because trial recruitment of people in an earlier stage of PD will be necessary (11, 112), population-based studies are essential to provide prediction algorithms based on environmental risk factors, biomarkers and polygenic risks (53, 55, 56).

Second, prevention or disease-modification trials need sufficient information on the strength of the causal relationship between a risk factor and PD in order to choose appropriate interventions. Insights from population-based studies, especially regarding environmental and lifestyle interventions, are highly important herein. Advanced statistical methods can be used to increase confidence in the causal association between risk factors and PD. A promising example includes Mendelian randomization, in which a genetic variation is used as a natural randomization to study the causal relation between a risk factor, for instance serum urate, and the risk of PD (113, 114). Mendelian randomization, however, still relies on several important assumptions, including the assumption that the genetic variant influences the outcome only through the exposure of interest (113). Furthermore, most current Mendelian randomization studies are performed in case-control settings (115), with the possibility of the previously reported survivor bias (116). Therefore, in order to draw causal conclusions from observational data, it remains crucial to combine the results of several types of studies with different potential sources of bias (117, 118).

### From Trials to Population-Based Studies

Theoretically, observational study designs are a direct extension of the underlying principles of a randomized controlled trial (RCT) (18, 119). Nevertheless, in practice, many important design principles of RCTs are not made explicit in the analyses of observational studies (119–121). Explicitly specifying the protocol of an intended (hypothetical) trial, including eligibility criteria and treatment strategies, and resembling this protocol as closely as possible in observational studies is called emulating a target trial (120, 122).

In other fields, emulating target trials in observational studies has already shown promising results, which more closely resemble real trial results than usual methods deployed in observational research (121, 123, 124). For example, large observational studies suggested that postmenopausal hormone users had a reduced risk of coronary heart disease, whereas the Women's Health Initiative randomized trial found a greater risk in women assigned to hormone replacement therapy than in those assigned to placebo (121). Hernán et al. mimicked the design of the randomized trial in a large observational study and showed that the discrepancies in results could largely be explained by differences in distribution of time since menopause and length of follow-up (121). This example illustrates that common differences in the results of observational and randomized studies could be accounted for when applying the design principles of RCTs in observational studies.

The approach of emulating target trials is novel in PD research, but it provides promising perspectives. Emulating a target trial helps observational studies to ask the most meaningful questions (125), to clarify assumptions underlying the analyses and to overcome common biases, such as immortal time bias and confounding bias (119, 120, 125, 126). In addition, emulating a target trial could be useful when trials cannot be performed because they are too costly, unethical or not timely (120). Results from observational studies and trials could also be combined, for instance to translate the results to a population with less restrictive eligibility criteria or to more long-term outcome measures instead of surrogate endpoints (119, 121). In the coming years, collaboration between trial initiatives and observational population-based studies is essential to make the first steps toward PD prevention.

## Conclusion

In this perspective we have explicated the role of population-based studies in unraveling the heterogeneous nature of pre-diagnostic PD. Population-based studies provide unique design advantages for studying pre-diagnostic PD, but they remain relatively sparse (2, 8, 13, 14). It is therefore pivotal that population-based studies join forces. Data sharing is essential to study interactions between genetic and environmental risk factors and to determine subgroup differences in the preclinical and prodromal phase of PD. Moreover, knowledge sharing and collaboration initiatives are required to bridge gaps between different study designs and to facilitate the transition toward prevention and disease-modification trials (20, 47).

## Data Availability Statement

The original contributions presented in the study are included in the article/supplementary material, further inquiries can be directed to the corresponding author/s.

## Author Contributions

LD drafted the manuscript. AB and MI critically revised the manuscript for intellectual content. All authors contributed to the article and approved the submitted version.

## Conflict of Interest

The authors declare that the research was conducted in the absence of any commercial or financial relationships that could be construed as a potential conflict of interest.
